# Pharmacogenetics to Personalize Dosing of Tricyclic Antidepressants in Major Depressive Disorder: A Randomized Clinical Trial (PITA study)

**DOI:** 10.1192/j.eurpsy.2023.275

**Published:** 2023-07-19

**Authors:** S. Ter Hark, N. Vos

**Affiliations:** Psychiatry, Radboudumc, Nijmegen, Netherlands

## Abstract

**Introduction:**

Tricyclic antidepressants (TCAs) have an important role in pharmacotherapy of Major Depressive Disorder (MDD). TCA dosing is aimed at achieving a concentration in the therapeutic window. Due to inter-individual differences in activity of Cytochrome P450 (CYP) 2D6 and 2C19, (enzymes involved in TCA metabolism) patients require an individualized dose. Currently, finding of a suitable dosage is a process of trial-and-error, including multiple drug concentration measurements and dose adjustments. A starting dose based on a patient CYP2D6 and CYP2C19 metabolizer phenotype may contribute to achieving a therapeutic concentration more quickly, possibly resulting in higher treatment efficacy and the occurrence of fewer adverse effects.

**Objectives:**

We aimed to study whether genotype-informed dosing in TCAs leads to faster attainment of therapeutic plasma concentrations, a higher reduction of depression severity symptoms and less adverse effects.

**Methods:**

We conducted a double-blind randomized clinical trial, in which patients (18-65 years) diagnosed with severe MDD and eligible for treatment with a TCA were randomized in two treatment arms: The intervention arm (Pharmacogenetics Informed Treatment; PIT) and standard treatment (Treatment As Usual; TAU). Patients were treated with nortriptyline, clomipramine or imipramine for seven weeks and depressive symptom severity and adverse effects were monitored weekly. The primary outcome measure, time needed to attain a therapeutic drug concentration, was analyzed using Kaplan-Meier analyses. The secondary outcome measures, reduction in depressive symptoms and adverse effects, were analyzed with mixed model linear regression.

**Results:**

In total, we randomized 111 patients. The PIT group (n=56) reached a therapeutic plasma concentration significantly faster (Kaplan Meier, X2 (1) = 4.3, p=0.039) compared to TAU (n=55), especially for patients treated with nortriptyline. On average depressive symptoms decreased relatively more in PIT than in TAU, although this was not significantly different: F(6) = 0.45, p = 0.84. The severity of adverse events during the study period differed significantly between PIT and TAU (F(6)=3.10, p=0.008). PIT experienced fewer adverse effects than TAU, especially in the last two weeks of the study.

**Conclusions:**

Genotype-informed dosing leads to faster attainment of therapeutic TCA concentrations and does not lead to more adverse effects. Although we were not able to demonstrate that genotype-informed dosing leads to a significantly better response, we conclude that pharmacogenetics can contribute to the optimization of pharmacological treatment. Future research may focus on subgroups for which pharmacogenetics may be of great value, such as specific antidepressants or patients with an abnormal pharmacogenetic profile.

**Disclosure of Interest:**

None Declared

